# Transcriptome Analysis of the Chicken Follicular Theca Cells with miR-135a-5p Suppressed

**DOI:** 10.1534/g3.120.401701

**Published:** 2020-09-08

**Authors:** Yan Zhou, Jie Liu, Qiuxia Lei, Haixia Han, Wei Liu, Tang Cunwei, Fuwei Li, Dingguo Cao

**Affiliations:** * Institute of Poultry Science, Academy of Agricultural Sciences of Shandong Province, Jinan, 250023, Shandong, China; † Poultry Breeding Engineering Technology Center of Shandong Province, Jinan, 250023, Shandong, China; ‡ Pingyi Yike poultry Co., Ltd, Linyi, 276000, Shandong, China

**Keywords:** *Gallus gallus*, ovarian theca cells, gga-miR-135a-5p, transcriptome sequencing

## Abstract

As a class of transcription regulators, numerous miRNAs have been verified to participate in regulating ovary follicular development in chickens (*Gallus gallus*). Previously we showed that gga-miR-135a-5p has significant differential expression between high and low-yield chicken ovaries, and the abundance of gga-miR-135a-5p is significantly higher in follicular theca cells than in granulosa cells. However, the exact role of gga-miR-135a-5p in chicken follicular theca cells is unclear. In this study, primary chicken follicular theca cells were isolated and then transfected with gga-miR-135a-5p inhibitor. Transcriptome sequencing was performed in chicken follicular theca cells with or without transfection. Differentially expressed genes (DEGs) were analyzed using bioinformatics. A dual-luciferase reporter assay was used to verify the target relationship between gga-miR-135a-5p and predicted targets within the DEGs. Compared with the normal chicken follicle theca cells, 953 up-regulated and 1060 down-regulated genes were detected in cells with gga-miR-135a-5p inhibited. The up-regulated genes were significantly enriched in Gene Ontology terms and pathways involved in cell proliferation and differentiation. In chicken follicular theca cells, Krüppel-like factor 4 (KLF4), ATPase phospholipid transporting 8A1 (ATP8A1), and Complexin-1 (CPLX1) were significantly up-regulated when the expression of gga-miR-135a-5p was inhibited. In addition, KLF4, ATP8A1, and CPLX1 confirmed as targets of gga-miR-135a-5p by using a dual-luciferase assay *in vitro*. The results suggest that gga-mir-135a-5p may involve in proliferation and differentiation in chicken ovarian follicular theca cells by targeting KLF4, ATP8A1, and CPLX1.

MicroRNAs (miRNAs) are a class of small noncoding RNAs of about 18-24 nucleotidesin length (Lee *et al.* 1993; Bartel 2004) that function as regulators in post-transcriptional gene expression by targeting sequence-specific sites in the 3′-untranslated region (3′-UTR) of mRNA (Grosshans and Slack 2002; Ambros and Chen 2007; Krol *et al.* 2010). Studies indicate that miRNAs play key roles in ovarian follicular development and function, including the formation of primordial follicles, follicular recruitment and selection, follicular atresia, oocyte-cumulus cell interaction, granulose or theca cell function, and luteinization (Hawkins and Matzuk 2010; Hasuwa *et al.* 2013; Kang *et al.* 2013; Maalouf *et al.* 2015; Zhang *et al.* 2019). Mouse miR-145 and miR-181a (Yan *et al.* 2012; Zhang *et al.* 2013), bovinelet-7 families *et al.* (Salilew-Wondim *et al.* 2014), buffalo miR-210 (Shukla *et al.* 2018), porcine miR-26b *et al.* (Lin *et al.* 2012) and chicken miR-107 (Miao *et al.* 2016) were all validated to be involved in granulosa cell proliferation, apoptosis and other cell function. In ovary theca cells, studies have showed that miRNAs also play an important role in cell function. In bovine, miR-640 and miR-526b* (Sohel *et al.* 2013), and bta-miR-335 (Gebremedhn *et al.* 2015) were proved to express more abundant in theca cells. Several predicted miRNA target interactions miR-155/miR-222-ETS1miR-199a-5p-JAG1, miR-155-MSH2and miR-199a-5p/miR-150/miR-378-VEGFA in theca cells were putatively involved in follicular atresia (Donadeu *et al.* 2017). Another study showed that abundance of MIR-221 was 66.sixfold greater (*P* < 0.001) in TCs than in GCs in bovine large follicles, and thecal MIR-221 expression is increased by FGF9 (Robinson *et al.* 2018). In sheep, northern analyses showed that the expression of miR-199a-3p, miR-125b, miR-145, miR-31, miR-503, miR-21 and miR-142-3p intheca cells were higher than those in granulose cells (McBride *et al.* 2012). In woman, two miRNAs-miR-92a and miR-92b were validated to be significantly downregulated in theca cells and might be involved in the pathogenesis of PCOS (Lin *et al.* 2015). In addition, miR-26a-5p was verified to facilitate theca cell proliferation in chicken ovarian follicles by targeting TNRC6A (Kang *et al.* 2017; Wu *et al.* 2019).

MiR-135a were proved to be overexpressed in GCs from PCOS patients, a study showed that miR-135a repressed ESR2 expression in GCs, which further inhibited CDKN1A expression, promoted GC proliferation and repressed GC apoptosis (Song *et al.* 2019). Furthermore, another finding indicates that miR-135a promotes apoptosis and the DNA damage response in GCs in PCOS, likely via VEGFC signaling (Wei *et al.* 2020). To our knowledge, the function of gga-miR-135a-5p in chicken follicular theca cells has not been reported. Our previous study showed that gga-miR-135a-5p was differentially expressed, with a fold-change of 8.93, in high compared low-yield ovaries of a Chinese indigenous chicken breed, and it was expressed significantly higher in follicular theca cells than in granulosa cells (unpublished). Therefore, the overall results indicated that miR-135a-5p may play an important role in chicken follicular theca cells.

In this study, we first transfect the gga-miR-135a-5p inhibitor into chicken follicular theca cells. RNA sequencing was performed for transcriptome analysis using the Illumina HiSeq sequencing platform. Adual-luciferase report assay was used to verify the regulatory relationship between miR-135a-5p and the predicted differentially expressed gene (DEG) targets. We present evidence that gga-miR-135a-5p is involved in the biological function of ovarian follicular development. This study providesa scientific basis for a mechanism of gga-miR-135a-5p regulation in the follicular development of poultry.

## Materials and Methods

### Ethics statement

All experimental procedures were approved by the Animal Care Committee of the Academy of Agricultural Sciences, ShandongProvince, Ji’nan, China. The care and use of experimental animals were carried out in accordance with the Directory Proposals on the Ethical Treatment of Experimental Animals, established by the Ministry of Science and Technology (Beijing, China).

### Birds and tissue harvest

Three single-comb white Leghorn hens were selected randomly from Shandong Poultry Breeding and Engineering Technology Research Center to be used in this study. All birds were reared in an environmentally controlled house. Fresh water and feed were provided according to the Feeding Standard established by the Ministry of Agriculture (Beijing, China). At 40 weeks of age, the F1-F5 follicles were removed carefully and placed in pre-cooled phosphate-buffered saline (PBS) for the next step of theca cell culture.

### Theca cell culture and transfection

The F1-F5 follicle theca layers were separated according to Kang *et al.* (Kang *et al.* 2017). The isolated theca layers were minced to 1mm^3^ pieces and digested with collagenaseII (w/v, 0.2%, Gibco, Grand Island, New York, USA) at 37° for 30 min. Remove the supernatant, the cell precipitation digested with collagenaseII (w/v, 0.2%, Gibco) at 37° for 30 minagain. The dispersed theca cells were filtrated with a sterilized 200-mesh filter and then centrifuged at 1800× rpm for 10 min. The cell precipitations were washed two times with cell culture medium containing M199 (HyClone, Logan, Utah, USA) supplemented with 10% (v/v) fetal bovine serum (Gibco) and 1% (v/v) penicillin-streptomycin solution (Solarbio, Beijing, China). The cells were then seeded in 24-well plates at a density of 2×10^5^ per well and cultured at 37° in an atmosphere of 95% air and 5% CO2. The number of viable cells (>90%) was estimated using Trypan blue.

When the cells reached approximately 60–70% confluence, they were transfected with gga-miR-135a-5p inhibitor and a miRNA inhibitor negative control (NC) with Lipofectamine2000 (Invitrogen, Carlsbad, CA, USA) and Opti-MEM (Gibco) according to the manufacturer’s instructions. Each transfection was performed at least in triplicate. The transfection efficiency was confirmed by real-time quantitative reverse transcription PCR (qRT-PCR). The primers used for gga-miR-135a-5p amplification were as follows: loop primer 5‘-GTCGTATCCAGTGCAGGGTCCGAGGTATTCGCACTGGATACGACTCACATA -3′; forward: 5‘- GCGAGAGGTATGGCTTTTTATTCC-3′, reverse: 5‘-GTGCAGGGTCCGAGGT -3′.U6 was used as an internal control: loop primer: 5′-GTCGTATCCAGTGCAGGGTCCGAGGTATTCGCACTGGATACGACCGTATACA -3′; formard: 5′- CACTGGGCCATGCTAATCTTCTC -3′; reverse: 5′- GTGCAGGGTCCGAGGT -3′.

### RNA extraction, cDNA library construction and sequencing

A total of five samples, including two groups of normal chicken follicular theca cells groups T07 and T08 (NG) and 3 groupstransfected with gga-miR-135a-5p inhibitor T10, T11, and T12 (TG) were used for sequencing. The total RNA of each sample was extracted with Trizol (Aidlab, Beijing, China) according to the manufacturer’s instructions. RNA integrity and concentration were checked using an Agilent 2100 Bioanalyzer (Agilent Technologies, Inc., Santa Clara, CA, USA). The mRNA was isolated by using NEBNext Poly (A) mRNA Magnetic Isolation Module (E7490, NEB, Ipswich, MA, USA). The cDNA library was constructed following the instructions of the NEBNext Ultra RNA Library Prep Kit for Illumina (NEB, E7530) and NEBNext Multiplex Oligos for Illumina (NEB, E7500). In brief, the enriched mRNA was fragmented into approximately 200nt RNA inserts, which were used to synthesize the first-strand cDNA and the second cDNA. End-repair/dA-tail and adaptor ligation were performed on the double-stranded cDNA. Suitable fragments were isolated by AgencourtAMPure XP beads (Beckman Coulter, Inc.), and enriched by PCR amplification. Finally, the constructed cDNA libraries were sequenced on a flow cell using an Illumina HiSeq sequencing platform.

### Transcriptome analysis using reference genome-based reads mapping

Low quality reads, such as adaptoronly, unknown nucleotides > 5%, or Q20 <20% (percentage of sequences with sequencing error rates <1%), were removed using aperl script. The clean reads filtered from the raw reads were mapped to the chicken genome (*Gallus gallus*, Galgal 4.75) using Tophat2 (Kim *et al.* 2013) software. The aligned records from the aligners in BAM/SAM format were further examined to remove potential duplicate molecules. Gene expression levels were estimated using fragments per kilobase of exon per million fragments mapped (FPKM) values by Cufflinks software (Trapnell *et al.* 2010).

### Identification of DEGs

DESeq and Q-value were employed and used to evaluate differential gene expression between cells expressing gga-miR-135a-5p (NG) and those with gga-miR-135a-5p inhibited (TG). After that, gene abundance differences between those samples were calculated based on the ratio of the FPKM values. The false discovery rate (FDR) control method was used to identify the threshold of the *P*-value in multiple tests in order to compute the significance of the differences. Here, only genes with an absolute value of log2 fold change ≥1 and FDR significance score <0.01 were used for subsequent analysis.

### Functional annotation

The Database for Annotation, Visualization, and Integrated Discovery (DAVID v6.7) was used to annotate the Gene Ontology (GO) and Kyoto Encyclopedia of Genes and Genomes pathways of the DEGs. The GO includes biological process, molecular function, and cellular component categories. An online software analysis tool (http://www.lc-bio.cn/overview/12?tools=GO_BarPlot) was used to plot GO functional classification of the unigenes with a GO term hit to view the distribution of gene functions. Finally anonline software analysis tool (http://www.lc-bio.cn/overview/14?tools=KEGG_BarPlot) was used to map the enriched pathways associated with the DEGs.

### Quantitative real-time PCR validation

To confirm the differential expression results, we conducted quantitative RT-PCRin a LightCycler 96 Real-Time PCR system (Roche, Switzerland) using a PrimeScript RT reagent Kit with a gDNA Eraser (Takara, Japan) and TB Green Premix Ex Taq II (TliRNaseH Plus, Takara, Japan) following the manufacturer’s directions. A total of 12 genes were used in qPCR to determinethe abundance of mRNAs. *β-actin* (Sangon Biotech, China) was used for normalization of the expression data. The relative mRNA expression level was calculated using the 2^-ΔΔCT^method.All the primers for qRT-PCR are exhibited in Table 1. Three independent replications for each sample were used and data are presented as means ± SD.

**Table 1:** Information of the primers used for quantitative real-time PCR validation

Genes	Tm(°C)	Sequences (5′-3′)	Products (bp)
**IFIT5**	60	F: GAAGAACCCAACAACCCAGA	127
		R: GTTTCAGTGCACGCTTCAGA	
**IFI6**	60	F:AAGCCGGTTTCACTTCCTCT	135
		R: CTTTGGCACCCATTTCTTGT	
**SERPINB2**	60	F:GCAGATCAGATGGCAAAGGT	131
		R: AGGCACGGTGATTTGATAGG	
**NSG1**	60	F: TTTCAGCGAGAAGAGCACAA	119
		R: TGACCACAACCTTGTCAGGA	
**GRK5**	60	F: TGCTCCTCACCAAAGATGTG	120
		R: CCAGCATTCCTGCTTCTAGC	
**CXCL12**	60	F: AGGGCCAACATTAAGCACCTC	110
		R: TTTAGCTTGGGATCAATGCACAC	
**FABP7**	60	F: GGGAACGTGACTAAGCCCACA	172
		R: TGTCTCCATCCAGGGTCACAAC	
**KLF4**	60	F: CTGCGGCAAGACCTACACCA	123
		R: AGTTCATCAGATCGGGCAAACTTC	
**ATP8A1**	60	F:TCTAACTGTGTGTTTGAAGGCTGGA	145
		R: ATCGGGTGCCATAGGGATGA	
**STK35**	60	F: GGATCGACCTGATGCCTTTGA	137
		R: CGTTGCACGTAATGGAGATGATG	
**FRMD4A**	60	F: AAGGACAACGCCACCATTGAG	102
		R: TGCAGCCAGTTCAAACACCAC	
**OASL**	60	F:AGATGTTGAAGCCGAAGTACCC	106
		R: CTGAAGTCCTCCCTGCCTGT	
**β-actin**	60	F: TAAGCGTGTTATCATCTC	86
		R: GGGACTTGTCATATTTCT	

### Dual-luciferase report assay

The 3′-UTR sequence of KLF4, ATP8A1, and CPLX1 harboring the gga-miR-135a-5p binding sites were amplified with the primers ATP8A1_WT, CPLX1_WT, and KLF4_WT (Table 2). The PCR products were cloned into the pmiR-RB-REPORT (Ribibio, China) vector to construct the wild-type plasmid, designated ATP8A1_WT, CPLX1_WT and KLF4_WT. The gga-miR-135a-5p binding sites were mutated in the WT vectors to construct the mutant luciferase reporter vectors designated ATP8A1_mut, CPLX1_mut, and KLF4_mut. Then, 293T cells were seeded into 24-well plates, and cotransfected with mimics or non-target control at a concentration of 50 nmol/L and 250 ng wild type or mutant luciferase reporter plasmids. After transfection for 48 hr, luciferase activities were measured using the Dual-GloLuciferase Assay System (Promega, USA).

**Table 2:** Primers for the construction of dual-luciferase reporter plasmid

Genes	Primers (5′-3′)	Products (bp)
ATP8A1	ATP8A1_WT _F: GCGGCTCGAGAGCACTTGTAGTTCTGATG	687
	ATP8A1_WT _R: AATGCGGCCGCACATAGCGACCACTTTCTG	
	ATP8A1_mut_F: TAGAACTGTTCGGTATAATGCACTAATATTGTTT	
	ATP8A1_mut_R: AGTGCATTATACCGAACAGTTCTACAATAAAGAA	
CPLX1	CPLX1_WT _F: GCGGCTCGAGTGAACAAGACCAAAGATAA	781
	CPLX1_WT- R _R: AATGCGGCCGCACAGAGAGAGAAAACAAGC	
	CPLX1_mut_F: TTTAGAGATTCGGTATTGAAATCAGACTGCAATA	
	CPLX1_mut_R: TGATTTCAATACCGAATCTCTAAAGATAGGTATG	
KLF4	KLF4_WT _F: GCGGCTCGAGATCACCTCGCCTTACACAT	1033
	KLF4_WT _R: AATGCGGCCGCTCAACCACTGACCAACATT	
	KLF4_mut_F: CCTTCTAATTCGGTATACAATGTTAAAAGAGGA	
	KLF4_mut_R: ACATTGTATACCGAATTAGAAGGAAGAAAAAAA	

Note: WT, wild type; MUT, mutant type.

### Data availability

The raw sequence data reported in this paper have been deposited in the Genome Sequence Archive (Genomics, Proteomics & Bioinformatics 2017) in Beijing Institute of Genomics (BIG) Data Center (Nucleic Acids Res 2019), Chinese Academy of Sciences, under accession number CRA001745 that are publicly accessible at http://bigd.big.ac.cn/gsa. Supplemental material available at figshare: https://doi.org/10.25387/g3.12899993.

## Results

### Chicken follicle theca cells transfected successfully with gga-miR-135a-5P inhibitor

**Figure 1:**
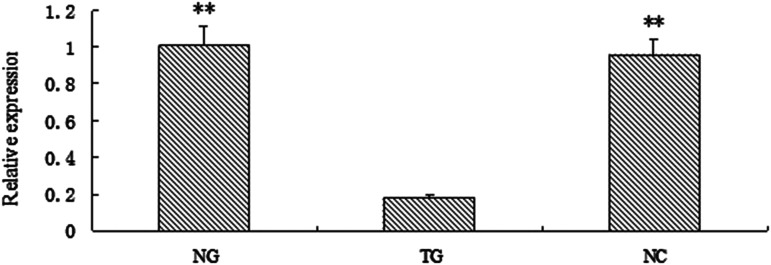
The relative expression level of gga-miR-135a-5p in NG, TG and NC. ** *P* < 0.01. NG: follicular theca cells; TG: inhibitor transfected cells; NC: negative control.

The expression level of gga-miR-135a-5p in chicken follicular theca cells, cells transfected with an inhibitor, and NC were detected by qRT-PCR, as shown in Figure 1, compared with the control and normal chicken follicular theca cells, the relative expression level of gga-miR-135a-5p decreased significantly in the cell inhibitor group (*P* < 0.01).

### RNA-seq and data analysis

After stringent filtration quality control by removing adaptors and low quality reads, 34,217,686, 43,452,332, 31,645,116, 32,409,966, and 35,082,796 clean reads were obtained in the T07, T08, T10, T11, and T12 samples, respectively, of which 75.62%, 76.59%, 75.48%, 75.46%, and 76.30% were mapped to the chicken (*Gallus gallus*) reference sequence, respectively.

### RNA expression and differential analysis

**Figure 2:**
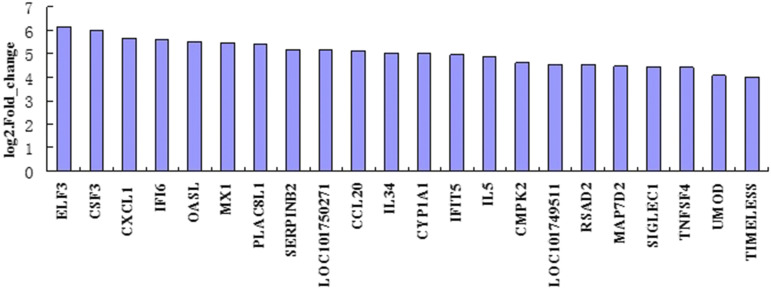
The expression level of the top 22 up-regulated genes with the most significant differential expression greater than fourfold between cells with normal and inhibited expression of gga-miR-135a-5p.

**Figure 3:**
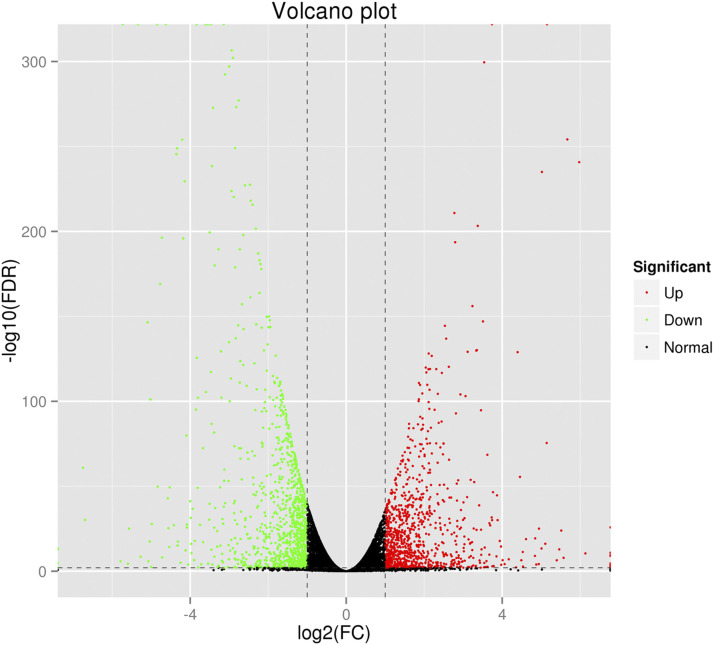
Volcano plot of differentially expressed genes in cells with normal and inhibited expression of gga-miR-135a-5p. The X-axis represents log2 (FC) and Y -axis represents –log10 (FDR). The green dots indicate the down-regulated genes, the black dots indicate the genes with no significant differences, and the red dots indicate up-regulated genes.

**Figure 4:**
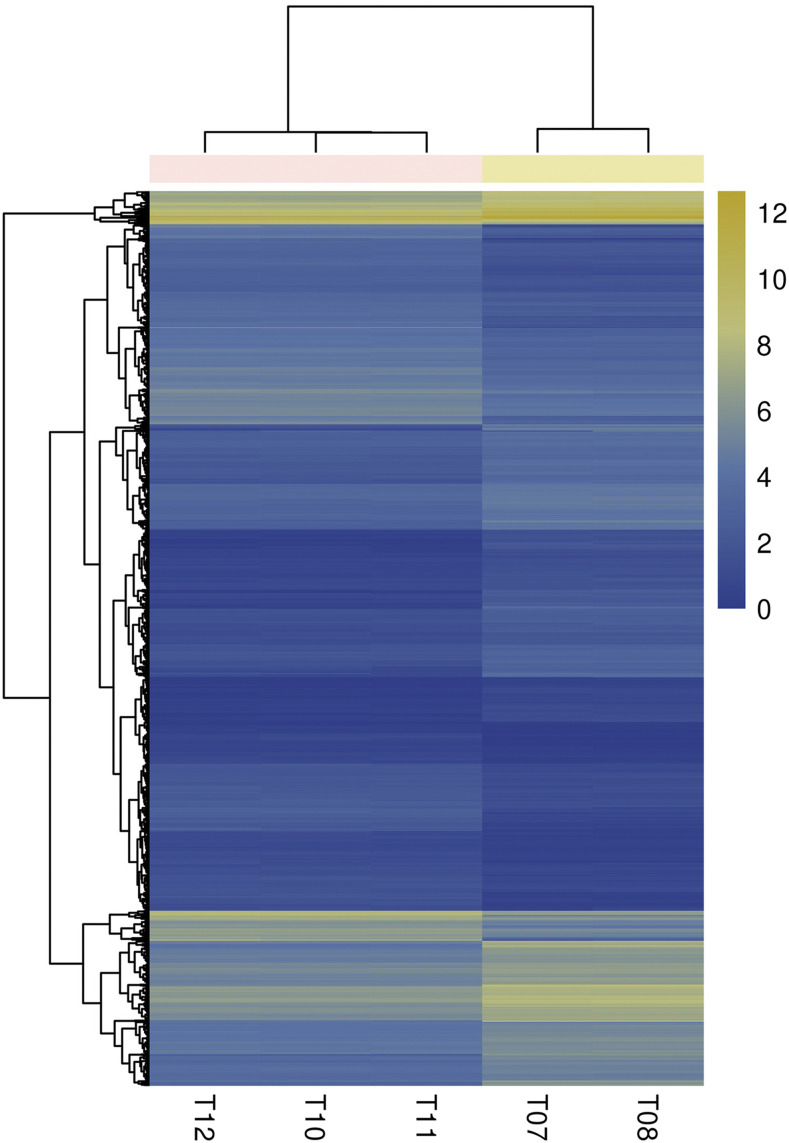
The heatmap of differentially expressed genes across all samples via Illumina sequencing. The depth of the color represents the level of gene expression in samples. Normal group: T07 and T08; Transfected group: T10, T11, and T12.

Based on the filtering criteria of the gene abundance differences with an absolute value of log2 fold change ≥1and FDR significance score <0.01, 2013 genes were found to express with a significant difference between cells with gga-miR-135a-5p expression (T07 and T08) and cells with gga-miR-135a-5p inhibited (T10, T11, and T12), while 953 genes were significantly up-regulated, and 1060 genes were significantly down- regulated in cells with gga-miR-135a-5p inhibited (Additional file: Table S1). Only 22 known up-regulated genes showed differences greater than log2 fold change ≥4 between the groups (Figure 2).The volcano plot of DEGs in different groups is shown in Figure 3. Hierarchical clustering analysis of DEGs was performed, and the result of the heatmap is shown in Figure 4.

### Functional annotation of DEGs

**Figure 5:**
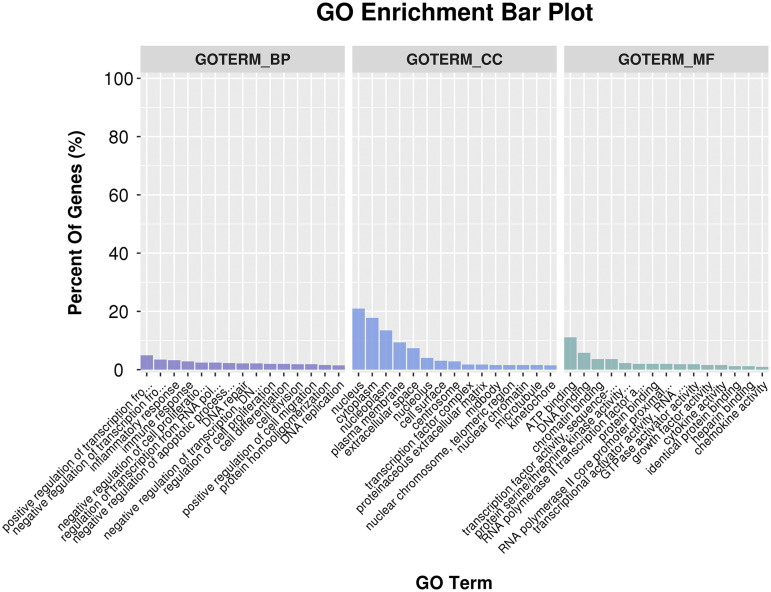
GO annotation of differentially expressed genes,The y-axis on the left indicates the percentage of genes in each term, the x-axis indicates the enriched GO terms.

The GO enrichment analysis for up-regulated DEGs showed that a total of 125 terms were enriched in biological processes, including cell proliferation, cell differentiation, cell division, regulation of transcription from RNA polymerase II promoters, etc. Among these, 39 terms were preferentially enriched in cell components, such as nucleus, cytoplasm, nucleoplasm, and plasma membrane and 34 terms were enriched in molecular functions including: ATP binding, DNA binding, and transcriptional activator activity (Figure 5).

**Figure 6:**
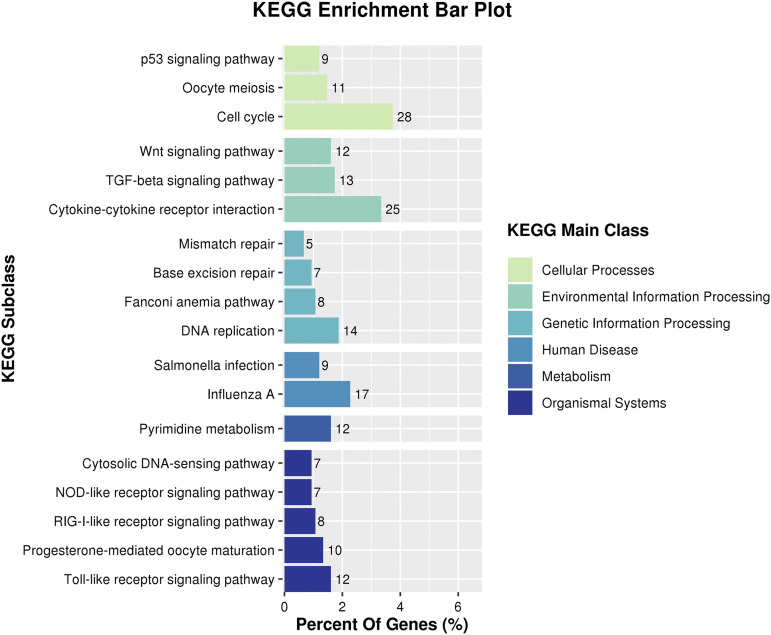
KEGG annotation of up- regulated differentially expressed genes.

The KEGG analysis showed that18 terms were enriched (Figure 6). The up-regulated DEGs were preferentially enriched in pathways associated with cellular functions such as the cell cycle (cell division), cytokine-cytokine receptor interaction (cell growth, differentiation, and cell death), the TGF-beta signaling pathway (cell proliferation, apoptosis, differentiation, and migration), Wnt signaling pathway (cell-fate specification, progenitor-cell proliferation, and the control of asymmetric cell division),and p53 signaling pathway (cell differentiation).

### Quantitative real-time PCR validation

**Figure 7:**
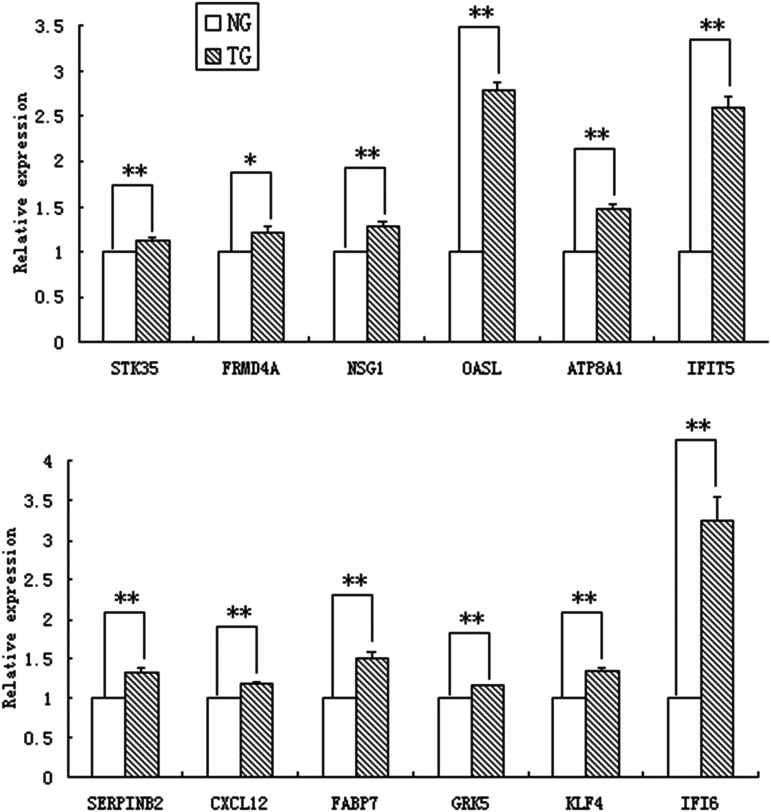
Relative expression of 12 DEGs between cells with normal (NG) and inhibited (TG) expression of gga-miR-135a-5p.

To verify the RNA-seq data, 12 DEGs were selected randomly for relative expression analysis by quantitative RT-PCR (Figure 7). The results showed that the relative expression of Serpin family B member 2 (SERPINB2), C-X-C motif chemokine ligand 12 (CXCL12), fatty acid-binding protein 7 (FABP7), G protein-coupled receptor kinase 5 (GRK5), Krüppel-like factor 4 (KLF4), interferon-alpha inducible protein 6 (IFI6), serine/threonine kinase 35 (STK35), neuron specific gene family member 1 (NSG1), 2′-5′-oligoadenylate synthetase-like (OASL), ATPase phospholipid transporting 8A1 (ATP8A1) and interferon-induced protein with tetratricopeptide repeats 5 (IFIT5) in transfected cells were significantly higher than those in normal cells (*P* < 0.01), whereas the relative expression ofFERM domain-containing 4A (FRMD4A) between the two groups was different with *P* < 0.05. The results of quantitative real-time PCR validation were consistent with RNA-seq data, which confirms the reliability of the RNA-seq results.

### KLF4, ATP8A1, and CPLX1are target genes of gga-miR-135a-5p

**Figure 8:**
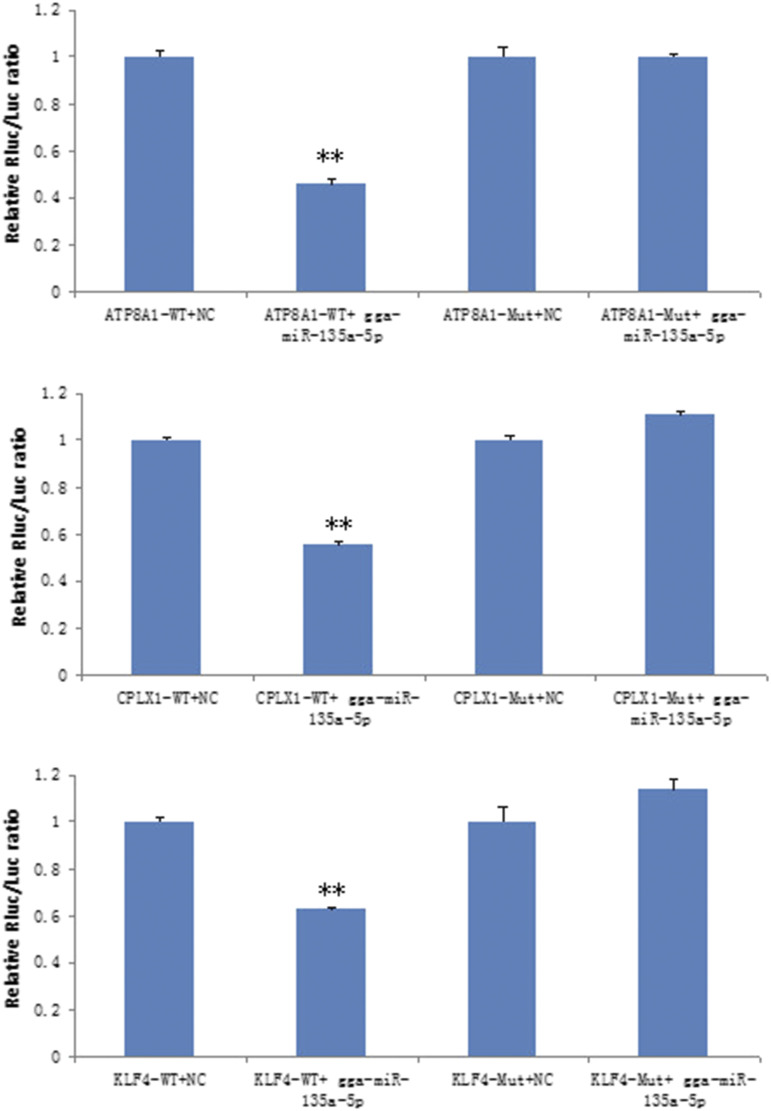
gga-miR-135a suppresses the expression of KLF4, ATP8A1, and CPLX1 in 293T cells. **P < 0.01.

To further reveal the regulated mechanism of gga-miR-135a-5p in follicular theca cells in chicken, two online algorithms (TargetScan and Pictar) were used to identify the target genes of miR-135a-5p, the up-regulated DEGs KLF4, CPLX1, and ATP8A1 appeared in both databases. Although, the RNA-seq data indicated that the expression level of KLF4, CPLX, and ATP8A1 were significantly up-regulated with the gga-miR-135a-5p inhibitor, to confirm their relationship *in vitro*, we cotransfected the wild-type or mutant luciferase reporter vectors of each gene and corresponding mimics or a non-target control into 293T cells. The dual-luciferase reporter assay results showed that miR-135a-5p decreased the activity of luciferase with wild-type KLF4, ATP8A1 and CPLX1 (Figure 8). After the mutation of predicted target sites, the reported fluorescence in mutant vectors recovered. These results suggest that miR-135a-5p may directly regulate gene expression by binding to sites on the 3′-UTR of the three target genes, KLF4, ATP8A1, and CPLX1.

## Discussion

The function of miR-135a in human disease has been widely studied (Tang *et al.* 2014; Zhang *et al.* 2017). It has been found to play an important role in several diseases including epithelial ovarian cancer (Tang *et al.* 2014), diabetes (Agarwal *et al.* 2013), malignant glioma (Zhang *et al.* 2016), sepsis (Zheng *et al.* 2017), and endometriosis lesions (Petracco *et al.* 2019). In addition, miR-135a-5p was found to be critical for exercise-induced adult neurogenesis (Pons-Espinal *et al.* 2019), by controlling NCX1 expression, miR-135a modulates cardiomyocyte automaticity, Ca^2+^ extrusion, and arrhythmogenic Ca^2+^loading/spontaneous Ca^2+^ release events to contribute to proarrhythmic remodeling after Complete atrioventricular block (Duong *et al.* 2017). As an important regulatory factor, miR-135ais involved in the regulation of 3T3-L1 preadipocyte differentiation and adipogenesis through the activation of canonical Wnt/β-catenin signaling by directly targeting Apc (Chen *et al.* 2014). In chicken, lncRNA–gga–miR-135a - mRNA interactions may promote the adipogenic differentiation of chicken preadipocytes (Chen *et al.* 2019). Our study provides evidence of gga-miR-135a-5p involvement in chicken follicular theca cells cytopoiesis.

We performed transfection with gga-miR-135a-5p inhibitor to suppress its expression in chicken follicular theca cells, and then performed transcriptome sequencing. Compared with the normal follicular theca cell group, 2013 differential expression genes composed of 953 up-regulated genes and 1060 down-regulated genes were identified. Bioinformatic analyses showed that the up-regulated DEGs were enriched in the TGF-β signaling pathway (gga04350), p53 signaling pathway (gga04115), and Wnt signaling pathway (gga04310), which are known to be involved in follicular development (Xu *et al.* 2018; Du *et al.* 2018). The up-regulated DEGs BMP2, BMP4, TGFβ2, TGFβ3, and BAMBI are enriched in the TGF-β signaling pathway and have a promotive function in granulosa cell proliferation, follicle survival, and prevention of premature luteinization and/or atresia (Schmid *et al.* 1994; Souza *et al.* 2002; Knight and Glister 2006). THBS1, CCNB2, and CDK1 are enriched in the p53 signaling pathway and have been found to play a role in granulosa cell proliferation in beef cattle (Dias *et al.* 2013), humans (Tremblay and Sirard 2017) and ovine species (Talebi *et al.* 2018). The Wnt family has been implicated in follicular development, and its components the up-regulated DEGs WNT2B, AXIN2, SFRP4, WNT6, NFATC2, WNT5B, and BAMBI have also been found to play important roles in ovarian follicle development (Li *et al.* 2014; Hatzirodos *et al.* 2014; Gupta *et al.* 2014; Drake *et al.* 2003; Chen *et al.* 2012). Thus, gga-miR-135a-5p may regulate the follicular theca cell development through the important signaling pathways mentioned above.

Bioinformatics analysis showed that the KLF4, CPLX1, and ATP8A1 DEGs were the target genes of gga-miR-135a-5p. The expression of KLF4, CPLX1, and ATP8A1 was significantly up-regulated in the gga-miR-135a-5p inhibitor group, and the direct binding relationships between them were further validated by a dual-luciferase reporter assay.

Krüppel-like factor 4 (KLF4) belongs to the KLF family of transcription factors, and exerts important biological effects on cellular proliferation, differentiation, and apoptosis (Ghaleb *et al.* 2005; Natesampillai *et al.* 2008; Black *et al.* 2001) in various cells types. Combined LH and IGF-I stimulation increased KLF4 mRNA expressed in porcine ovarian granulosa cells (Natesampillai *et al.* 2008). An H_2_O_2_-induced *in vitro* model and a 3-nitropropionic acid (NP)-induced *in vivo* model of mouse ovarian oxidative stress showed that miR-145 protects granulosa cells against oxidative stress-induced apoptosis by targeting KLF4 (Xu *et al.* 2017).The regulatory function of KLF4 on rat preovulatory granulosa cells also has been confirmed by Hyeonhae and Jaesook (Choi and Roh 2019).They found that KLF4 increases the susceptibility of preovulatory granulosa cells to apoptosis by down-regulating Bcl-2, and promotes an LH-induced cell cycle exit. Interestingly, a directly targeted gene of gga-miR-135a-5p, KLF4, was significantly enriched in the negative regulation of cell proliferation (GO:0008285) and regulation of cell differentiation (GO:0045595), which indicated that gga-miR-135a-5p may up-regulate the expression of KLF4 to regulate the proliferation and differentiation of chicken follicular theca cells.

CPLX1 belongs to a highly conserved complexin protein family and encodes a neuronal protein(Fernandez and Dittman 2009). Studies have shown that Cplx1(−/−) mice have profound ataxia that limits their ability to perform co-ordinated motor tasks, and have pronounced deficits in social behaviors (Drew *et al.* 2007). The CPLX1 variants were involved in patients with ID, developmental delay, and myoclonic epilepsy (Brose 2008; Kielar *et al.* 2012; Redler *et al.* 2017). In chicken, there is a lack of data for CPLX1, it was only found to be correlated to earlobe color in Rhode Island Red chickens (Nie *et al.* 2016). Our results suggest that CPLX1 was not only strongly linked with the neurodevelopmental function of the neuronal cell body (GO:0043025), regulation of neurotransmitter secretion (GO:0046928), and synaptic growth at the neuromuscular junction (GO:0051124) as previously reported, but also involved in the biological process of transmembrane transport (GO:0055085). We speculated that the gga-miR-135a-5p may participate in the regulation of the transmembrane transport function of chicken follicular theca cells by targeting CPLX1.

ATP8A1 is a member of the P4-ATPases subfamily. In mammalian cells, ATP8A1 has been implicated in the translocation of phospholipids (Daleke and Lyles 2000). In Chinese hamster ovary cells, the phospholipid flippase complex of ATP8A1 and CDC50A was found to play a major role in cell migration (Kato *et al.* 2013). Also, ATP8A1 was verified to play a role in regulating the growth and mobility of non-small-cell lung cancer cells (Dong *et al.* 2016). In this study, as confirmed by previous studies, ATP8A1 was enriched in the biological processes involved in phospholipid transport, such as: phospholipid-translocating ATPase activity (GO:0004012), phospholipid transport (GO:0015914) and amino phospholipid transport (GO:0015917). It was also enriched in the biological processes associated with cell development, such as positive regulation of multicellular organism growth (GO:0040018) and negative regulation of cell proliferation (GO:0008285). Interestingly, ATP8A1 was the direct target of gga-miR-135a-5p, this implies a regulation mechanism of gga-miR-135a-5p in chicken follicular theca cells by down-regulating ATP8A1.

In conclusion, gga-miR-135a-5p can directly target the 3′-UTR of KLF4, CPLX1, and ATP8A1 genes to inhibit their expression in chicken follicular theca cells. The data show that gga-mir-135a-5p may play an important role in regulating chicken ovarian follicular theca cells development. Our findings eliminate a gap in the knowledge of gga-miR-135a-5p regulation in chicken follicular theca cells. The exact regulation of apoptosis or proliferation by gga-miR-135a-5p in chicken follicular theca cells and the pathways involved, will be the focus of our future work.
